# Tumour necrosis factor-α stimulates HIV-1 replication in single-cycle infection of human term placental villi fragments in a time, viral dose and envelope dependent manner

**DOI:** 10.1186/1742-4690-3-36

**Published:** 2006-06-23

**Authors:** Anfumbom KW Kfutwah, Jean-Yves Mary, Marie-Anne Nicola, Sandra Blaise-Boisseau, Françoise Barré-Sinoussi, Ahidjo Ayouba, Elisabeth Menu

**Affiliations:** 1 Laboratory of Virology, Centre Pasteur du Cameroun, Yaoundé, Cameroon; 2 INSERM U717, Université Paris 7, Hôpital St Louis, Paris, France; 3 Plate-forme d'Imagerie Dynamique, Institut Pasteur, Paris, France; 4 Unité Régulation des Infections Rétrovirales, Institut Pasteur, Paris, France

## Abstract

**Background:**

The placenta plays an important role in the control of *in utero *HIV-1 mother-to-child transmission (MTCT). Proinflammatory cytokines in the placental environment are particularly implicated in this control. We thus investigated the effect of TNF-α on HIV-1 expression in human placental tissues *in vitro*.

**Results:**

Human placental chorionic villi fragments were infected with varying doses of luciferase reporter HIV-1 pseudotypes with the R5, X4-Env or the vesicular stomatitis virus protein G (VSV-G). Histocultures were then performed in the presence or absence of recombinant human TNF-α. Luciferase activity was measured at different time points in cell lysates or on whole fragments using *ex vivo *imaging systems.

A significant increase in viral expression was detected in placental fragments infected with 0.2 ng of p24 antigen/fragment (P = 0.002) of VSV-G pseudotyped HIV-1 in the presence of TNF-α seen after 120 hours of culture. A time independent significant increase of viral expression by TNF-α was observed with higher doses of VSV-G pseudotyped HIV-1. When placental fragments were infected with R5-Env pseudotyped HIV-1, a low level of HIV expression at 168 hours of culture was detected for 3 of the 5 placentas tested, with no statistically significant enhancement by TNF-α. Infection with X4-Env pseudotyped HIV-1 did not lead to any detectable luciferase activity at any time point in the absence or in the presence of TNF-α.

**Conclusion:**

TNF-α in the placental environment increases HIV-1 expression and could facilitate MTCT of HIV-1, particularly in an inflammatory context.

## Background

*In utero *transmission of the human immunodeficiency virus type 1 (HIV-1) is one of the routes through which infants acquire HIV-1 from their mothers [1]. However, the placenta forms an efficient barrier between maternal and foetal circulations and plays a crucial role in the regulation of mother-to-child transmission (MTCT) of HIV-1. The trophoblasts are the placental cells in direct contact with maternal circulation. It has been observed that transmission of HIV-1 between T lymphocytes and trophoblasts is possible [2,3] and that cell-to-cell contact between HIV-1 infected peripheral blood mononuclear cells (PBMCs) and cells of trophoblast origin led to the passage of virus from the apical to the basolateral side of a trophoblast barrier reconstituted *in vitro *[4]. Soluble factors such as RANTES and MIP-lβ decrease viral passage through this placental trophoblast barriers inoculated with HIV-1 infected cells while TNF-α and IL-8 increase it [5].

On the other hand, isolated primary trophoblasts or malignantly transformed human cell lines of trophoblast lineage are highly resistant to infection with cell-free HIV-1 particles. This resistance has been shown to be bypassed when HIV-1 envelope are substituted by the vesicular-stomatitis virus G protein in the HIV-1 pseudotypes used to infect trophoblast cells [6]. Vidricaire and colleagues observed that cell-free viruses are able to enter with high efficiency into trophoblast cell lines [7]. However viral entry is mediated in large part through endosomal vesicles which ends up in the degradation of a majority of these viral particles but the initial presence of HIV-1 within the endosomes seems to be mandatory for infection to take place in these cells [8]. Production of fully infectious HIV-1 particles by trophoblast cells was only seen following treatment with TNF-α or IL-1 and subsequent co-culture with indicator cells bearing a reporter gene. This confirmed a previous study showing that cytokines, notably TNF-α, stimulate replication of HIV-1 in trophoblast cells [9].

Few studies have been carried out on term placental tissue *ex vivo *[10]. Maury et al. showed that term placental tissue can support a low HIV-1 replication when infected with cell-free laboratory viral strain used at high doses and after coculture with indicator PBMCs. No study to the best of our knowledge addressed the effect of TNF-α on HIV-1 replication within the placental tissue. Since TNF-α is known to be expressed in the placenta environment especially during viral [11,12] or parasitic infections [13,14], HIV-1 placental tissue infection was studied in the presence or absence of recombinant human TNF-α using the placental histoculture system recently described [14] and a single-cycle viral replication system.

## Results

### Placental histocultures replicate VSV-G pseudotyped HIV-1 in a time and viral dose dependent manner in the absence of TNF-α stimulation

As shown on Table 1, only background level of luciferase activity was detected after 48, 96 and 120 h of histoculture when the envelope deficient (delta-Env) pseudotyped HIV-1 was used.

When placental fragments were infected by the VSV-G pseudotyped HIV-1, a dose dependent replication was observed with an increase of luciferase activity overtime. At the lowest dose of VSV-G pseudotyped HIV-1 used (0.2 ng of p24 antigen/placental fragment), after an increase at 96 h, the luciferase activity dropped at 120 h. In contrast with higher doses, the increase remained up to 120 h.

**Figure 1:**
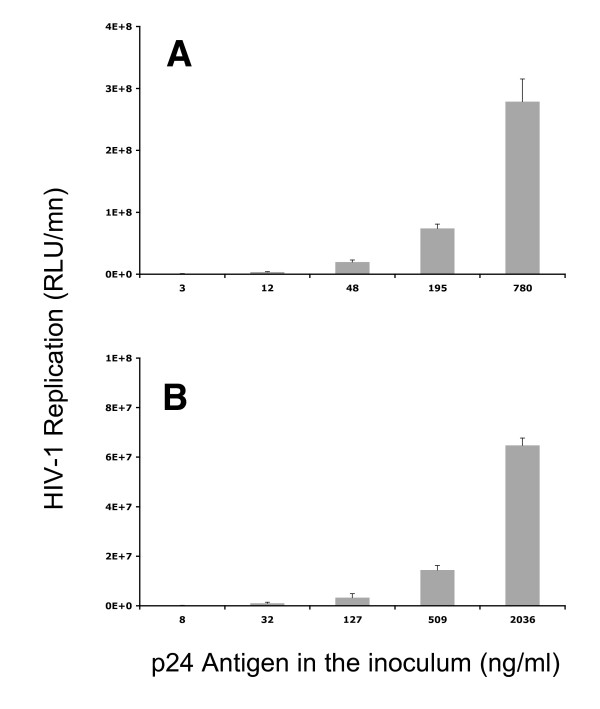
**Titration of Ba-L and HXB2 pseudotyped HIV-1 on HeLa P4P cells**. 10^4 ^HeLa P4P cells/well/100 μl were seeded in 96 flat-bottomed well plates. Infections were performed 24 hours later. Cells were lysed 72 hours post infection with 100 μl of lysis buffer (Promega, Madison USA). 100 μl of commercially available luciferase substrate (Promega, Madison, USA) were added to 20 μl of each lysate and then luciferase activity read with a luminometer (Lumat LB 9501, Berthold) as relative light units (RLU) per minute. A: Ba-L pseudotyped HIV-1; B: HXB2 pseudotyped HIV-1.

No luciferase activity was observed in preliminary results obtained after infection kinetics of placental fragments (48–120 h post infection) with R5 and X4 pseudotyped HIV-1 despite the fact that control cells (HeLa P4P) were highly infected by both pseudotypes (Figure 1). We therefore left placental fragments after overnight contacts with R5 and X4 pseudotyped viruses in culture for longer periods (120–216 h post infection). For the R5 envelope pseudotyped HIV-1, a modest increase of replication was observed at 168 h post infection at the highest concentration used (50 ng of p24 antigen/placental fragment) for 3 placentas out of 5 tested in the whole range of kinetics. These data show that despite the extended culture periods, the placental fragments remained resistant to infection with HIV-1 pseudotypes bearing X4 (HXB2) envelopes even at concentration as high as 100 ng of p24 antigen/placental fragment. It should be noted that the very same placentas were readily infected by the HIV-1 pseudotype bearing the VSV-G envelope.

### TNF-α significantly increases the replication of VSV-G pseudotyped HIV-1 in placental histocultures

In all experimental conditions, the dose effect of TNF-α (5 vs 50 ng/ml) did not vary with the duration of histoculture (absence of interaction between TNF-α and duration of histoculture) and TNF-α effect was homogeneous across doses. Therefore, the TNF-α effect is presented globally as mean ratio, whatever the TNF-α dose used.

**Figure 2:**
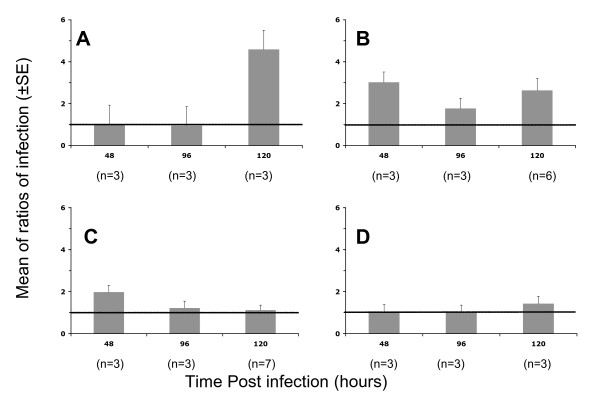
**Infection with VSV-G or delta-Env pseudotyped HIV-1 ofplacental chorionic villi in the absence or presence of TNF-α**. Viral pseudotypes were left in contact with placental fragments overnight. After viral contact, fragments were cultured in medium supplemented with (grey histograms) or without (horizontal black line) TNF-α and cultures stopped at 48, 96, 120 hours post-infection. Fragments were homogenized and luciferase activity read from tissue lysate. Results are presented as ratio (mean ± SE) of luciferase activity with TNF-α stimulation to activity without stimulation in three to seven (n) placentas from different donors. A: VSV-G pseudotyped HIV-1 at 0.2 ng of p24/placental fragment; B: VSV-G pseudotyped HIV-1 at 2 ng of p24/placental fragment; C: VSV-G pseudotyped HIV-1 at 20 ng of p24/placental fragment; D: delta-Env pseudotyped HIV-1 at 20 ng of p24/placental fragment.

When the envelope deficient (delta-Env) pseudotyped HIV-1 was used, luciferase activity was not modified by TNF-α after 48, 96 and 120 h of histoculture duration (mR of 1.16 ± 0.20, p = 0.45) (Figure 2D).

**Figure 3:**
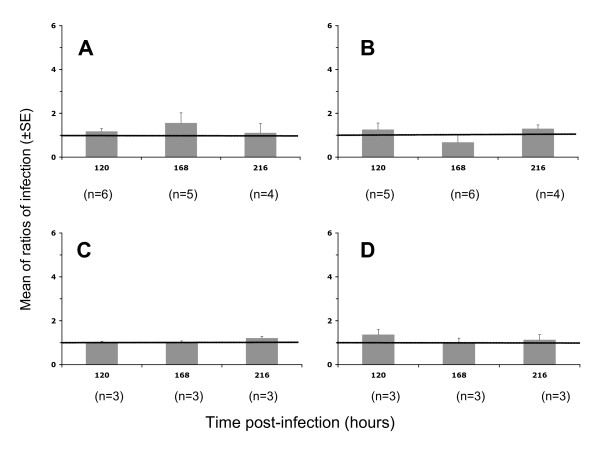
**Infection with R5 or X4 envelope pseudotyped HIV-1 of placental chorionic villi in the absence or presence of TNF-α**. Viral pseudotypes were left in contact with placental fragments overnight. After viral contact, fragments were cultured in medium supplemented with (grey histograms) or without (horizontal black line) TNF-α and cultures stopped at 120, 168 or 216 hours post-infection. Fragments were homogenised and luciferase activity read from tissue lysate. Results are presented as ratio (mean ± SE) of luciferase activity with TNF-α stimulation to activity without stimulation in three to seven (n) placentas from different donors. A: R5 (Ba-L) pseudotyped HIV-1 at 20 ng of p24/placental fragment; B: R5 (Ba-L) pseudotyped HIV-1 at 50 ng of p24/placental fragment; C: X4 (HXB2) pseudotyped HIV-1 at 50 ng of p24/placental fragment; D: X4 (HXB2) pseudotyped HIV-1 at 100 ng of p24/placental fragment.

For the lowest dose of VSV-G pseudotyped HIV-1 (0.2 ng of p24 antigen/placental fragment), the effect of TNF-α on the viral replication was highly significant with a mR of 4.59 ± 0.91 (p = 0.002) after 120 hours postinfection and was not observed at earlier time points with a mR of 1.04 and 1.01 after 48 and 96 h, respectively (Figure 2A). At higher viral doses, 2 and 20 ng of p24 antigen/placental fragment, an increase of viral replication was observed in the presence of TNF-α with a global mR of 2.19 ± 0.28 (p = 0.001, Figure 2B) and 1.41 ± 0.19 (p = 0.05, Figure 2C), respectively. For the other pseudotypes tested, R5 (Figure 3A and 3B) or X4 (Figure 3C and 3D) envelope pseudotyped HIV-1, TNF-α did not significantly increase the viral replication in the placental histocultures, whatever the time point or the viral dose used, with a global mR of 1.08 ± 0.14 (p = 0.56) and 1.11 ± 0.07 (p = 0.15), respectively.

**Figure 4:**
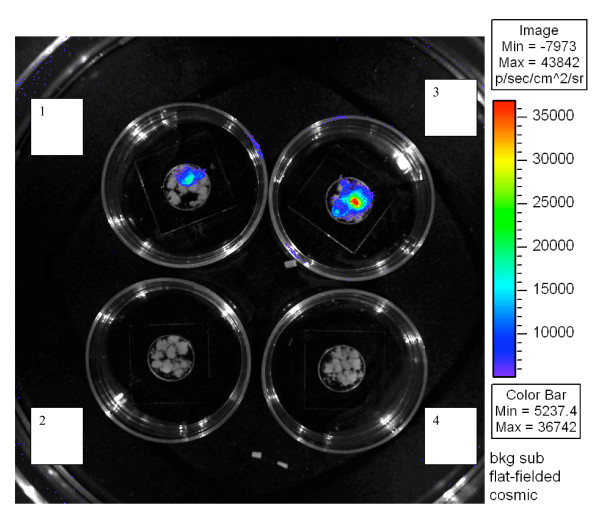
***Ex vivo *bioluminescence imaging of HIV-1 infected placental chorionic villi stimulated or not with TNF-α**. Placental chorionic villi were placed in contact with pseudotyped HIV-1 overnight. After the addition of luciferine for 4 h at 37°C, the tissues were analysed with the Xenogen system. Infection of chorionic villi with 2 ng of p24/fragment of VSV-G pseudotyped HIV-1 in the absence (well 1) or presence of 5 ng/ml of TNF-α (well 3). Infection of chorionic villi with 20 ng of p24/fragment of delta-Env pseudotyped HIV-1 in the absence (well 2) or presence of 5 ng/ml of TNF-α (well 4).

With the Xenogen system, infection with the VSV-G pseudotyped HIV-1 was also detected, demonstrated by an increase in the bioluminescence emitted from placental fragments stimulated with TNF-α compared to those without TNF-α treatment. In a representative case shown in Figure 4, bioluminescence values without TNF-α were 31,501 photons/sec, and 93,449 photons/sec with 5 ng/ml of TNF-α. Using this system, we observed that not all placental fragments were detectably infected with the VSV-G pseudotyped HIV-1 despite the fact that they all underwent the same treatments. No bioluminescence was detected in the xenogen system when the delta-Env pseudotyped HIV-1 was used with or without TNF-α stimulation.

## Discussion

This study demonstrates the fact that human term placental chorionic villi can be infected and stimulated to replicate HIV-1 when VSV-G pseudotyped HIV-1 is used. When R5-Env pseudotyped HIV-1 is used, a low level of transient infection can be detected for some of the placentas tested. In contrast when X4-Env pseudotyped HIV-1 is used, no infection is detected.

Previous studies have shown that malignantly transformed human cell lines of the trophoblast lineage are resistant to cell-free HIV-1 pseudotypes bearing the R5 and X4 envelopes [6]. In this study we demonstrate that the human placental chorionic villi are not permissive to X4-Env pseudotyped HIV-1 and display limited permissivity to R5-Env pseudotyped HIV-1 as exemplified by the low luciferase activity values observed even in the presence of the highest dose of TNF-α. This lack of activation of the HIV-1 replication with TNF-α contrasts with the results obtained with choriocarcinoma cell lines by Vidricaire and colleagues who reported an increase of luciferase activity in these cells after infection with R5 or X4-Env pseudotyped HIV-1 in the presence of TNF-α [7]. These apparent contradictory findings might be explained by different experimental conditions. Our study was performed on human term placental tissue and not on cell lines. R5 or X4-Env pseudotyped HIV-1 viruses were used in a dose range of 20 to 100 ng of p24 antigen to infect human placental fragments in contrast to the 250 to 400 ng of p24 antigen used by Vidricaire and colleagues to infect cells. Finally in our experiments, the TNF-α treated placental fragments were not co-cultured with indicator cells to amplify replication because we used non productive single-cycle replicating viral pseudotypes.

The fact that 3 placentas out of 5 were modestly and transiently infected by R5-Env pseudotyped HIV-1 might have some biological relevance in the context of HIV-1 MTCT. Indeed, HIV-1 MTCT occurs mainly with R5 viruses. Additionally, 5–10% of infants born to HIV infected mothers are infected *in utero *in the absence of any prophylaxis [15]. Thus, there might be a difference in the susceptibility of some placentas to cell-free R5 virus infection and transmission.

The chorionic villi are made up of several cell types such as syncytiotrophoblasts, cytotrophoblasts, Hofbauer cells and stromal cells which has been shown to express variable levels of surface CD4, CXCR4 and CCR5 [16-19]. The expression of CD4 on the surface of trophoblasts is still a matter of debate, however even if they express CD4, they do not produce detectable viral particles when infected with fully competent infectious cell-free viruses [6].

A dose dependent infection was observed with VSV-G pseudotyped HIV-1 whose replication is increased by TNF-α. This implies that TNF-α would increase viral replication in the placenta when the initial resistance to cell-free HIV-1 infection *in vivo *is bypassed, after cell-to-cell infection for example. In fact, proinflammatory cytokines such as TNF-α and IL-8 have been shown to increase viral replication in different cell lines, isolated primary cells or cervical explant tissue [9,5,20]. Indeed, in our study, TNF-α appeared to be capable of stimulating HIV-1 replication in term placental chorionic villi with the various cell subpopulations present. It should be recalled here that VSV-G pseudotyped HIV-1 are able to infect any cell type, regardless of receptor and coreceptor surface expression, due to its amphotropic nature. It has been previously described that the mechanisms by which TNF-α enhance HIV-1 replication occurred via the NF-kB pathway [21]. TNF-α activates HIV-1 LTR-driven transcriptional activity in choriocarcinoma cell line [7] and isolated primary trophoblast cells [9]. Such a mechanism might also occur in placental chorionic villi. The fact that the TNF-α stimulation of HIV-1 replication is not dependent of the dose tested (5 and 50 ng/ml) is consistent with a recent finding demonstrating that the temporal profile of the NF-kB activity is invariant to a wide range of TNF-α doses going from 0.01 ng/ml up to 10 ng/ml [22].

It should be noted that the TNF-α effect is time and VSV-G pseudotyped HIV-1 concentration dependent. Hence, at 0.2 ng of p24 antigen of VSV-G pseudotyped HIV-1/fragment, the increase of replication by TNF-α is only observed at 120 h post-infection whatever the cytokine concentration. At 2 ng of p24 antigen of VSV-G pseudotyped HIV-1/fragment, the effect of TNF-α is only significant when data were analyzed globally for all the time points tested whereas at 20 ng of p24 antigen/fragment, the TNF-α effect is barely significant due to an already high basal replication without TNF-α (Table 1).

Furthermore, as shown by the Xenogen system (Figure 4), not all the placental fragments from the same experimental wells were infected with VSV-G-pseudotyped HIV-1 to detectable levels even in the presence of TNF-α. This suggests that the susceptibility to infection is not homogeneous for all the chorionic villi from a given placenta and thus explains the high variability observed between placentas. However, we cannot exclude that this non homogeneous infection is also due to the non saturating dose of pseudotypes used for this experiment. This result might also explain why there was no detectable stimulation of luciferase activity in the presence of TNF-α with the R5-Env pseudotyped HIV-1 while there was a modest and transient infection.

**Table 1:** Hiv-1 pseudotype replication in the human placental histoculture system in the absence of TNF-α stimulation

	Luciferase Activity (Relative Light Units/mn): mean (SE), n				
**Time post-infection:**	**48 h**	**96 h**	**120 h**	**168 h**	**216 h**
HIV-1/Delta-Env (20 ng*)	2,660 (356), 3	3,300 (1,637), 3	2,830 (911), 6		
HIV-1/VSV-G (0.2 ng*)	4,010 (1,224), 3	14,110 (9,591),3	5,420 (4,152), 3		
HIV-1/VSV-G (2 ng*)	14,520 (16,718), 3	77,880 (22,016), 3	93,640 (34,095), 7		
HIV-1/VSV-G (20 ng*)	213,190 (195,249), 3	1,266,430 (449,346), 3	1,397,280 (861,789), 3		
					
HIV-1/Ba-L (20 ng*)			2,562 (312), 6	2,535 (475), 5	2,670 (286), 4
HIV-1/Ba-L (50 ng*)			3,830 (3,612), 6	10,380 (7,478), 5	2,625 (772), 4
HIV-1/HXB2 (50 ng*)			2,620 (827), 3	3,010 (135), 3	2,210 (399), 3
HIV-1/HXB2 (100 ng*)			2,950 (458), 3	2,860 (255), 3	2,580 (1,046), 3

* : of p24 antigen

Altogether, these results highlight the subtle equilibrium between the viral load, the timing of cytokine expression and the regulation of viral replication within the placenta that might ultimately influence HIV-1 MTCT.

The demonstration that TNF-α could enhance HIV-1 replication in the placental tissue is important, since a shift in cytokine production towards a proinflammatory profile (notably TNF-α) has been associated with *Plasmodium falciparum *placental infections. [23,24,13]. This is of particular public health concern in regions of the world where HIV-1 and malaria are both endemic. Malaria has been shown to reach peak transmission periods during the raining seasons [25]. In an earlier work, a peak in HIV-1 MTCT was reported three months after the peaks of the rains in Cameroon [26], suggesting that the observed increase in HIV-1 MTCT could have been due to an increase of TNF-α in the placental environment as a result of malaria. The present study showing an increase of HIV-1 replication by TNF-α within the placental tissue supports this hypothesis. However direct evidence are still required before we can conclude that placental malaria is linked with an increase transmission of HIV-1 from mother-to-child via an increase of placental proinflammatory cytokines. As discussed in a recent review, several published studies show no, little or significant association of malaria with HIV-1 MTCT [27]. Some studies done on cohorts of pregnant women found no correlation between placental malaria and *in utero *HIV-1 MTCT [28]. Others studies have demonstrated that the effects of malaria on HIV-1 MTCT depends on placental malaria parasitemia [29].

## Conclusion

In summary, data presented here show, by using a single-cycle replication system, that TNF-α enhances HIV-1 replication in chorionic villi of the human placental tissue once viral entry is overcome, as seen with the VSV-G pseudotyped HIV-1. This implies that *in vivo *TNF-α would increase viral replication in the placenta when the initial resistance to cell-free HIV-1 infection is bypassed, after cell-to-cell infection for example. Altogether, these results highlight the subtle equilibrium within the placental environment between the viral load, the timing of proinflammatory cytokine expression and the regulation of viral replication that might ultimately influence HIV-1 MTCT.

## Methods

### Placenta collection and preparation

Term placentas from HIV-1 negative women were collected in accordance with French ethical guidelines after programmed caesarean section at the Antoine Béclère (Clamart, France) and Robert Debré (Paris, France) hospitals. Placentas were processed within 2 to 3 hours following collection to isolate chorionic villi. After extensive washes with normal medium (RPMI-1640 supplemented with 10% heat-inactivated fetal calf serum (FCS), 1% L-Glutamine and 1% Penicillin/Streptomycin), each placenta was cut to obtain several villi blocks of 2 to 3 mm. Blocks were then distributed in 12-well plates, nine villi blocks per well, and in triplicate wells. For each placenta, 63 to 144 blocks were prepared.

### Viral pseudotype preparation and infection of placental fragments

Luciferase reporter viruses were prepared as previously described [6], by co-transfecting 293T cells with the NL4-3 luciferase virus backbone (pNL-Luc E-R-) and VSV-G or HIV-1 R5 (Ba-L) or X4 (HXB2) env plasmids, with the aid of SuperFect (Qiagen, France). Each pseudotype preparation was tested for effective infection at different concentrations on HeLa P4P. Briefly, 10^4 ^cells/well/100 μl were seeded in 96 flat-bottomed well plates. Infections were performed 24 hours later. Cells were lysed 72 hours post infection with 100 μl of lysis buffer (Promega, Madison, WI, USA). 100 μl of commercially available luciferase substrate (Promega) were added to 20 μl of each lysate and then luciferase activity read with a luminometer (Lumat LB 9501, Berthold) as relative light units (RLU) per minute.

Different concentrations of the various viral pseudotypes in histoculture medium (RPMI 1640 supplemented with 1% Penicillin/Streptomycin, 1% L-Glutamine, 15% FCS, 0.1% gentamycin, 1% Fungizone, 1% non-essential amino acids, 1% sodium pyruvate -all from Gibco BRL Ltd, Paisley, Scotland, UK) were left in contact with the placental fragments in triplicate wells. Each set of triplicate placental wells contained one of the following pseudotype doses: 0.2 ng, 2 ng and 20 ng of p24 antigen/fragment of VSV-G-pseudotyped HIV-1, 20 ng and 50 ng of p24 antigen/fragment of R5-Env pseudotyped HIV-1 (Ba-L), 50 ng and l00 ng of p24 antigen/fragment of X4-Env pseudotyped HIV-1 (HXB2) and 20 ng of p24 antigen of the envelope deficient pseudotype (delta-Env pseudotyped HIV-1) used as a negative control for infection. All contacts were left overnight at 37°C in a 5% CO2 incubator.

### Placental histocultures and TNF-α stimulation

After overnight contact, the nine villi blocks from each well were transferred into 70 μm nylon cell strainers (BD Bioscience, Belgium) and rinsed three times in PBS (Gibco BRL Ltd, Paisley, Scotland, UK). As previously described [14], the villi blocks from each set of triplicate wells were then placed on collagen sponge gels (Pharmacia & Upjohn company, Germany) inside three different wells soaked in one case with 3 ml of plain histoculture medium and in the two other wells soaked in 3 ml of histoculture medium containing recombinant human TNF-α (R&D systems, Deutschland) in final concentrations of 5 and 50 ng/ml respectively.

### Luciferase activity quantification by luminometer or by *ex vivo *imaging system

Histocultures in the presence or absence of recombinant human TNF-α were stopped after 48, 96, 120, 168 or 216 hours post-infection. All nine fragments on each sponge were pooled inside a 14 ml round bottom tube containing 500 μl of lysis buffer (Promega, Madison USA) and homogenised with a homogeniser (Ultraturax, X620 CAT M-Zipperer GmbH, Germany). 100 μl of lysed tissue supernatants was mixed with 100 μl of commercially available substrate (Dual-Luciferase Reporter Assay System, Promega, Madison, USA) and then luciferase activity read with a luminometer (Lumat LB 9501, Berthold) as relative light units (RLU) per minute. For each type of pseudotype (VSV-G, Ba-L, HXB2 or negative control), the different doses of pseudotype and 3 to 4 different durations of histoculture were studied on the same placenta, all without and with the two doses of TNF-α.

We adapted a complementary *ex vivo *imaging system, (Xenogen IVIS100 and data analyzed by the Living Image Software, Xenogen Corp., Alameda, CA, USA), to visualize and measure the effect of TNF-α on HIV-1 replication in the placenta. Placental fragments were left overnight in contact with 2 ng of p24 antigen/fragment of VSV-G pseudotyped HIV-1 or 20 ng of p24 antigen/fragment of delta-Env pseudotyped HIV-1, then placed on collagen sponges with 0 or 5 ng/ml of TNF-α for 120 hours. The fragments were then collected and placed in contact with 1 μM luciferine (Promega) for 4 hours at 37°C, then the bioluminescence of placental fragments was detected in the Xenogen system and the photons per second from a constant region of interest (ROI) were summed.

### Statistical analysis

For each experimental condition, type and dose of pseudotype and duration of histoculture, luciferase activity was measured without and with 5 or 50 ng/ml of TNF-α on three to seven different placentas from different donors. Results of the luciferase activity without TNF-α stimulation are presented globally as mean ± se. To take into account the fact that the activities without and with stimulation were evaluated on the same placentas, the effect of each dose of pseudotype was expressed as the ratio of the activity with each dose of TNF-α to the activity without TNF-α in the same experimental condition, noted ratio of infection, R. Then, for each type and dose of pseudotype, these ratios were analysed through analysis of variance using a factorial design including 2 factors, TNF-α dose (5 or 50 ng/ml) and duration of histoculture (either 48, 96, 120 hours, or 120, 168, 216 hours), and 3 to 4 replicates of placentas. For the duration of histoculture of 120 and 168 hours, 5 to 7 replicates were available for some type of pseudotype. First, it was tested if TNF-α dose effect varied with the duration of histoculture (interaction between TNF-α dose and duration of histoculture). Second, if no interaction was evidenced, the homogeneity of TNF-α effect across doses was tested independently of the duration of histoculture, whereas, in presence of interaction, the homogeneity of TNF-α effect across doses was tested for each duration of histoculture. Finally, the TNF-α dose effect was tested, either globally if homogeneity was evidenced or for each dose otherwise, by comparing the mean of the corresponding ratios to 1, using the residual error derived from the analysis of variance. The TNF-α dose effect was expressed as the mean of the corresponding ratios (mR) and its standard error (mR ± SE). Therefore a mR of 4.0 for a given dose of TNF-α means that the luciferase activity was multiplied on an average by 4 after stimulation when compared to no stimulation.

## Competing interests

The author(s) declare that they have no competing interests.

## Authors' contributions

AKWK has conceived and performed all the histoculture experiments and drafted the manuscript. JYM participated in the design of the study and performed the statistical analysis. MAN contributed to the experiments with the Xenogen system. SBB has optimized the conditions to produce the different viral pseudotypes and has titrated them. FBS contributed to the design of the study and discussion of the results. AA contributed to all the steps of the study and helped to draft the manuscript. EM conceived the study and participated in its design and coordination and to draft the manuscript.
